# The effects of cardiac drugs on human erythrocyte carbonic anhydrase I and II isozymes

**DOI:** 10.1080/14756366.2020.1781844

**Published:** 2020-06-22

**Authors:** Onur Argan, Kübra Çıkrıkçı, Aybike Baltacı, Nahit Gencer

**Affiliations:** a Department of Cardiology, Faculty of Medicine, Balikesir University, Balikesir, Turkey; b Department of Chemistry, Science and Art Faculty, Balikesir University, Balikesir, Turkey

**Keywords:** Cardiac drugs, carbonic anhydrase, enzyme inhibition

## Abstract

Cardiovascular diseases are the leading cause of mortality worldwide. In recent years, the relationship between carbonic anhydrase inhibitors and atherosclerosis has attracted attention. In this study, we aimed to determine the *in vitro* effects of 35 frequently used cardiac drugs on human carbonic anhydrase I (hCA I) and II (hCA II). The inhibitory effects of the drugs on hCA I and hCA II were determined with both the hydratase and esterase methods. The most potent inhibitors observed were propafenone (hCA I: 2.8 µM and hCA II: 3.02 µM) and captopril (hCA I: 1.58 µM and hCA II: 6.25 µM). Isosorbide mononitrate, propranolol, furosemide, and atorvastatin were also potent inhibitors. The inhibitor constant, *K*_i_, value from the Lineweaver–Burk plot for propafenone was 2.38 µM for hCA I and 2.97 µM for hCA II. The tested cardiac drugs showed potent *in vitro* inhibition of the hCA I and II isozymes. Especially, in patients with atherosclerotic heart disease, these drugs may be preferred primarily due to the beneficial effects of carbonic anhydrase inhibition on atherosclerosis.

## 1. Introduction

Carbonic anhydrases (CAs, EC 4.2.1.1) are a group of enzymes that catalyse the transformation of carbon dioxide into bicarbonate. There are 15 human carbonic anhydrase (hCA) isoforms, and they all differ in their cellular/tissue localisation and enzymatic features^1^. Investigation of the properties of this family of enzymes is crucial for human health. Analyses of the inhibitory effects of different drugs on enzymes in the CA family are crucial for life. The cytosolic forms of these enzymes are CA-I and CA-II. The isozymes hCA I and hCA II are involved in respiration and acid–base homeostasis^2^. CAs’ catalysis of carbon dioxide hydration is of paramount importance for many physiological processes, which include pH and bicarbonate homeostasis, respiration, bone metabolism, and tumorigenesis.

In recent years, the relationship between CA and atherosclerosis has attracted attention. Atherosclerotic cardiovascular diseases are the leading cause of death worldwide^3^. Atherosclerotic lesions cause coronary artery disease, stroke, and peripheral artery disease. Coronary atherosclerotic plaque-related thrombotic occlusion results in acute myocardial infarction. Calcium accumulation is a crucial step in atherosclerosis^4^ and is associated with a higher risk for cardiovascular mortality due to coronary artery disease^5^. CAs have an important role in vascular calcification in humans^6–9^.

However, the potential role of CA inhibition related to cardiac drugs in atherosclerosis has not been sufficiently researched. Additionally, drug–enzyme interaction studies have recently gained great interest. Many adverse drug events may result from CA isozyme inhibition. Thus, CA has been increasingly studied by several scientists worldwide. Our laboratory has also specialised in this subject^10–13^. However, no studies have investigated the effects of some cardiac drugs on hCA activity. Therefore, the current study aimed to determine and compare possible alterations in the activity of hCA I and II caused by cardiac drugs.

## 2. Experimental part

### 2.1. Materials

Sepharose 4B, L-tyrosine, protein assay reagents, phenol red, and chemicals for electrophoresis were obtained from Sigma-Aldrich Co. All other chemicals were of analytical grade and obtained from either Sigma-Aldrich Co. or Merck. Medications were provided by a local pharmacy. The study was approved by the local ethics committee (Balikesir University Faculty of Medicine Clinical Research Ethical Committee, Balikesir, Turkey, Decision No. 2019/138 and Date: 09.10.2019).

### 2.2. Preparation of hemolysate and purification of enzyme

Blood samples (25 ml) from healthy human volunteers were collected. They were centrifuged at 1000*g* for 20 min at 4 °C, and the supernatant was removed. The packed erythrocytes were washed three times with 0.9% NaCl and then were hemolyzed in cold water. The pH of the hemolysate was adjusted to 8.5 with the solid Tris base. The 25-ml hemolysate was applied to an affinity column containing y Sepharose 4B-ethylene diamine-4-isothiocyanato-benzenesulfonamide^14^. CA isozymes were then eluted with 0.1 M NaCl/25 mM Na_2_HPO_4_ (pH 6.3) and 0.1 M CH_3_COONa/0.5 M NaClO_4_ (pH 5.6), which recovered hCA I and II, respectively.

### 2.3. Hydratase activity assay

CA activity was measured by the Maren method based on the determination of the time required for the pH to decrease from 10.0 to 7.4 due to CO_2_ hydration^15^. The assay solution was 0.5 M Na_2_CO_3_/0.1 M NaHCO_3_ (pH 10.0), and phenol red was added as the pH indicator. Carbon dioxide-hydratase activity was calculated in enzyme units (EU) with the following equation: *t*_0_ – *t*_c_/*t*_c_, where *t*_0_ and *t*_c_ are the times for the pH change of the nonenzymatic and the enzymatic reactions, respectively.

The drugs were triturated in a porcelain mortar pestle. Stock solutions of both drugs were prepared in dimethyl sulfoxide (DMSO). Different concentrations of the drugs were added to the enzyme activity determination medium with a total volume of 4.2 ml. The duration (in seconds) of the colour change from red to yellow in solution was measured in a 10-ml glass tube with a diameter of 1 cm. Control cuvette activity in the absence of the inhibitor was set at 100%. All compounds were tested in triplicate at each concentration used. For each inhibitor, an activity %–[inhibitor] graph was drawn (data not shown). The inhibitor concentration causing up to 50% inhibition (IC_50_) was determined from the graphs. It was determined that DMSO had no effect on the enzyme activities in any of the tested concentrations.

### 2.4. Esterase activity assay

CA activity was assayed by following the change in absorbance of 4-nitrophenyl-acetate to the 4-nitrophenylate ion at 348 nm over 3 min at 25 °C using a spectrophotometer (Perkin Elmer Lambda 25 model UV/VIS) according to a method described in the literature^16^. The inhibitory effects of the drugs on enzyme activities were tested under *in vitro* conditions. The *K*_i_ values were calculated from a Lineweaver–Burk^17^ plot and are shown in Table 1.

**Table 1.** IC_50_ values for cardiac drugs of human carbonic anhydrase I and II

COMPOUNDS	IC_50_ (µM) (hydratase) hCA I	IC_50_ (µM) (hydratase) hCA II	IC_50_ (µM) (esterase) hCA I	IC_50_ (µM) (esterase) hCA II
1. Ranolazine	503	544	600	645
2. Propafenone	228	235	2.80	3.02
3. Nifedipine	77.9	103	42.2	50.6
4. Diltiazem	624	477	232	325
5. Trimetazidine dihydrochloride	196	151	112	112
6. Carvedilol	61.2	79	20.2	46.4
7. Amlodipine	133	126	38.5	38.8
8. Captopril	121	131	1.58	6.25
9. Edoxaban	94	67	45.5	34.2
10. Doxazosin mesylate	228	215	303	417
11. Ivabradine	503	544	107	117
12. Ramipril	281	154	32.3	37.9
13. indapamide	276	198	90	124
14. Lercanidipine hydrochloride	201	159	294	423
15. Nebivolol hydrochloride	73.7	95.3	404	410
16. Bisoprolol hemifumarate	107	221	156	244
17. Isosorbide mononitrate	160	160	6.08	5.50
18. Candesartan cilexetil	185	271	342	177
19. Irbesartan	235	239	345	335
20. Rivaroxaban	220	204	189	244
21. Ticagrelor	168	195	54	52.1
22. Furosemide	120	178	6.23	4.95
23. Clopidogrel	250	364	94	103
24. Digoxin	74.9	88	23.1	32.8
25. Propranolol	1.77	1.44	1.58	6.25
26. Amiodorane hydrochloride	389	405	8.47	12.4
27. Metoprolol succinate	400	168	319	329
28. Acetylsalicylic acid	252	246	43	58.3
29. Atorvastatin	243	390	7.75	9.85
30. Prasugrel	155	205	330	429
31. Apixaban	88.4	110	384	386
32. Lacidipine	696	482	150	155
33. Dabigatran etexilate	113	116	15.3	15.3
34. Glyceryl trinitrate	220	272	x	x
35. Lidocaine HCl	19.6	2	x	x

## 3. Results and discussion

In this study, the *in vitro* effects of 35 frequently used cardiac drugs were investigated on human erythrocyte CA-I and CA-II. Affinity chromatography was used to purify hCA I and hCA II. SDS-PAGE was performed to determine the purity of the enzymes. The inhibitory effects of the drugs on hCA I and hCA II were determined with both the hydratase and esterase methods. The IC_50_ values were calculated from activity %–[I] graphs and are shown in Table 1. CA activity in the absence of a drug was set as 100% activity.

The most potent inhibitors were propafenone (hCA I: 2.8 µM and hCA II: 3.02 µM) and captopril (hCA I: 1.58 µM and hCA II: 6.25 µM). Isosorbide mononitrate (hCA I: 6.08 µM and hCA II: 5.5 µM), propranolol (hCA I: 1.25 µM and hCA II: 6.25 µM), furosemide (hCA I: 6.23 µM and hCA II: 4.95 µM), and atorvastatin (hCA I: 7.75 µM and hCA II: 9.85 µM) were also potent inhibitors. The *K*_i_ value from the Lineweaver–Burk plot for propafenone was 2.38 µM (non-competitive) for hCA I and 2.97 µM (non-competitive) for hCA II (Table 2).

**Table 2.** IC_50_ and *K*_i_ values of propafenone for hCA I and hCA II

Propafenone	IC_50_(µM) (Hydratase)	IC_50_(µM) (Esterase)	K_İ_ (µM) (Esterase)
hCA I	228	2.80	2.38
hCA II	235	3.02	2.97

Inhibition type is non-competitive inhibition.

CAs are drug-target enzymes. The inhibitors of these enzymes are important compounds for discovering new therapeutic agents and understanding enzyme–drug interactions in detail at the molecular level. Propafenone is a sodium channel-blocking antiarrhythmic drug. It works by blocking the activity of particular electrical signals in the heart that can cause arrythmias. It is used to restore a normal heart rhythm and maintain regular beats in supraventricular tachyarrhythmias^18^. The effect of these cardiovascular drugs on CA has not been studied; however, the effects of some of these agents on aldehyde oxidase were studied. Propafenone (2.5 µM), amlodipine (5.5 µM), and nifedipine (79% inhibition at 50 µM) have been shown to inhibit aldehyde oxidase to a certain extent^19^.

Atherosclerosis is a dynamic process that influences the aorta and its branches. Atherosclerotic plaques are a local thickening of the intima layer caused by cholesterol, hydroxyapatite and fibrous connective tissue accumulation, and proliferation of smooth muscle cells. The determinant step of athelerosclerosis is calcium precipitation, which traps cholesterol in the plaque precursor matrix, which includes calcium carbonate, triglycerides, lipoproteins, hydroxyapapatite, and calmodulin^4^. In addition, calcification is a marker of atherosclerosis and is used to detect atherosclerosis severity^20^.

Studies have shown that both CA-I and CA-II play an important role in the aetiology of vascular calcification as a component of atherosclerosis. In a study by Oksala et al., CA-II was highly expressed in human atherosclerotic plaques in patients with advanced atherosclerosis^21^.

Ayari et al. detected overexpression of CA-II in human atheroma plaques compared with healthy arterial tissue from the same patient in a comparative genome-wide microarray expression analysis. When patients were compared with each other, CA-II was overexpressed more than 1.7 fold in the atherosclerotic plaques^22^.

Yuan et al. studied the effects of CA on atherosclerosis in an atherosclerotic rat model and human aortic dissection and aneurysm caused by atherosclerosis. They demonstrated significantly higher CA-I levels in the animal atherosclerotic tissues, and CA-I levels increased in human atherosclerotic tissues. Moreover, methazolamide, a CA inhibitor, decreased CA-I levels in the animal model. These results suggest that the higher levels of CA-I are related to vascular calcification, and CA-I plays an important role in atherosclerosis and its progression^23^.

Atorvastatin belongs to a group of drugs known as statins, or HMG-CoA reductase inhibitors. Statins inhibit *de novo* cholesterol synthesis and decrease low-density lipoprotein (LDL). Their hypolipidemic effects result in stabilisation of atherosclerotic plaques, hence they are used for coronary and peripheral artery diseases^24^. Another study revealed that atorvastatin inhibited CYP3A4 enzyme activity in a concentration-dependent manner with an IC_50_ value of 48 µM^25^. Guidelines and studies have demonstrated that early use of statin therapy correlates with evident clinical benefits and reduced mortality in patients with atherosclerotic coronary artery disease^26–31^. Another study revealed that atorvastatin showed submicromolar–low nanomolar inhibition of the 15 hCA isoforms (hCA I–XIV)^32^.

A recent study by Yuan et al. demonstrated that CA-I expression and CA-I-mediated calcification are significantly associated with atherosclerosis progression, and methazolamide significantly reduces atherosclerosis and suppresses CA-I expression. According to the results of our study, CA may be responsible for the atherosclerosis-reducing effects of statins as a secondary pathway in addition to the LDL-lowering effect^23^.

Captopril is a competitive inhibitor of angiotensin converting enzyme (ACE). This enzyme is responsible for the conversion of angiotensin I to angiotensin II. Angiotensin II regulates blood pressure and is a key element of the renin–angiotensin–aldosterone system. Leppala et al. reported that captopril is an angiotensin I converting enzyme inhibitor with an IC_50_ value of 0.007 µM^33^. ACE inhibitors improve endothelial function, retard the progression of atherosclerosis, and reduce the risk of cardiovascular death, myocardial infarction, and stroke via ventricular remodelling and neurohumoral regulation. Therefore, these agents are recommended in the treatment of a wide range of diseases, including coronary artery disease, peripheral artery disease, heart failure, stroke, diabetes, and hypertension^34^. CA inhibition by captopril may be an additional pathway to prevent atherosclerosis.

Beta-blockers inhibit the sympathetic activity of beta-adrenergic receptors. Propranolol, a non-selective beta blocker, inhibits all beta receptors. This activity decreases cardiac contractility and heart rate. Propranolol is frequently used in the treatment of patients with ischaemic heart disease and hypertension. Sozzani et al. reported that propranolol is also an inhibitor of protein kinase C. The IC_50_ value of propranolol was approximately 150 µM^35^. In addition, propranolol has been reported to inhibit ATPase activity with an IC_50_ value of 4.4 mM^36^. Beta-adrenergic inhibitors significantly decrease the activity of CA^37^.

Furosemide is a loop diuretic that acts on the kidney. Furosemide inhibits the Na–K–2Cl cotransporter on the membrane of the epithelial cells of the thick ascending limb of the loop of Henle. The decreased sodium and chloride reabsorption results in diuresis and natriuresis. Furosemide is used to treat edoema in patients with heart failure^38^. Temel et al. reported that furosemide inhibits the activity of glucose-6-phosphate dehydrogenase with an IC_50_ of 0.526 mM^39^. Furosemide has been reported to contain primary sulfamoyl moieties and inhibit CA isoforms in the kidneys and other organs^40^.

Isosorbide mononitrate is a drug mainly used to treat angina pectoris. It relaxes the coronary arteries, thereby increasing the circulation in the ischaemic zone. Isosorbide mononitrate relaxes vascular smooth muscles through the formation of nitric oxide (NO). NO activates guanylyl cyclase, resulting in decreased blood pressure and relaxation of the veins and arteries. In our study, this drug inhibited CA isoenzymes at the micromolar level.

Acetazolamide is a classic CA inhibitor. It has been reported to show notable inhibitory effects on hCA I with an IC_50_ value of 5.8 nM. The IC_50_ values of cardiac drugs used in our study were higher than the IC_50_ value for acetazolamide in a previous study^41^.

## 4. Conclusions

In this study, the *in vitro* effects of 35 frequently used cardiac drugs were investigated on human erythrocyte CA-I and CA-II. In conclusion, the drugs showed potent inhibitory effects on hCA I and hCA II *in vitro*. Our results underline the potential of cardiac drugs to target atherosclerosis through hCAI-II inhibition. Especially, in patients with atherosclerotic heart disease, cardiac drugs that inhibit hCAs may be preferred primarily due to the beneficial effects of CA inhibition.
